# A conceptual analysis: generative artificial intelligence literacy in English as a foreign language writing

**DOI:** 10.3389/fpsyg.2026.1815271

**Published:** 2026-05-11

**Authors:** Guodong Jiang

**Affiliations:** School of Humanities and International Studies, Zhejiang University of Water Resources and Electric Power, Hangzhou, China

**Keywords:** critical evaluation, EFL writing education, English as a foreign language (EFL) writing, ethical consideration, generative artificial intelligence (GenAI) literacy, student writers

## Abstract

Generative artificial intelligence (GenAI) has transformed various aspects of English as foreign language (EFL) education and thus GenAI literacy in this field has attracted increasing academic attention. However, studies on GenAI literacy in EFL writing are still in nascent stages and GenAI literacy as a budding concept remains underexplored. This conceptual analysis aims to clarify the concept of GenAI literacy in EFL writing. Relevant literature indicated that definitions of GenAI literacy involve basic understanding, technical skills, critical evaluation, and ethical consideration about GenAI tools and outputs, with criticality as a core element for EFL writing. This analysis also aims to interpret prominent theories of GenAI literacy. It illustrated the socially-constructed foundation of GenAI literacy theories, covering affective, cognitive, behavioral, and ethical perspectives. This analysis developed a new framework of GenAI literacy after synthesizing theories of GenAI literacy in EFL writing. Based on the new framework consisting of five dimensions: understanding, intention, application, evaluation and ethics, the analysis then explored the pedagogical implications and research orientations for instructors, curriculum designers, policy makers, and scholars in GenAI-assisted EFL writing. This analysis can thus enrich their understanding of GenAI literacy. The new framework of GenAI literacy will enable them to effectively research and integrate GenAI into EFL writing education.

## Introduction

Generative artificial intelligence (GenAI) tools (e.g., ChatGPT) are widely and frequently utilized by student writers of English as a foreign language (EFL) or a second language (L2; Chen and Pi, 2026), who use them to generate essays, translate texts or improve language accuracy, clarity and cohesion of their own writing (Tang et al., 2025). However, they do not adequately understand GenAI's algorithmic principles and probabilistic nature and thus are not fully aware of inherent inaccuracies and biases in GenAI outputs (Tagare et al., 2025). Consequently, they often incorporate GenAI content into their writing without critical evaluation or ethical consideration (Yusuf et al., 2024), thus misrepresenting facts and plagiarizing others' work (Yan, 2023). All these issues can be attributed to their inadequate GenAI literacy, which involves knowledge, skills, ethical and critical use of GenAI tools (Wang et al., 2025).

In current literature, most studies on GenAI-aided EFL writing focus on learner perceptions of GenAI (e.g., attitude and motivation; Wang, 2025), the effects of GenAI on writing processes and outcomes (e.g., learning autonomy and writing quality; Yang et al., 2025), and learner behavioral patterns of using GenAI (e.g., prompting strategies and timing; Hwang et al., 2025). However, studies on GenAI literacy in EFL writing are still in nascent stages and GenAI literacy as a budding concept remains underexplored in this field (Ou et al., 2024). Therefore, given the importance of EFL students' GenAI literacy in writing, this conceptual analysis aims to illustrate key conceptualizations and theoretical frameworks as well as pedagogical implications in GenAI-assisted EFL writing.

## Key conceptualizations

### Artificial intelligence (AI) literacy

AI literacy is a multifaceted concept with multiple definitions. Some definitions highlight knowledge and understanding. For example, Kong et al. (2021) consider AI literacy as comprising three components, revolving around AI concepts:

“AI concepts, using AI concepts for evaluation, and using AI concepts for understanding the real world through problem solving.” (p. 2).

AI concepts in their conceptualization refer to technical knowledge and understanding of machine learning, decision trees, reasoning and prediction, patterns in data, and statistical inferences. Other definitions of AI literacy focus on ability and action without in-depth technical knowledge about AI. For instance,

“AI literacy is the ability to understand, interact with and critically evaluate AI systems and AI outputs.” (Lintner, 2024, p. 1).

Similarly, AI literacy is defined as competences to critically evaluate AI technologies, communicate and collaborate effectively with AI, and use AI as a tool online, at home, and in the workplace (Long and Magerko, 2020).

Therefore, AI literacy is not just technological proficiency (i.e., understanding and using AI tools). Instead, it involves ethical considerations: assessing whether the functions, designs and algorithms of AI align with ethical principles (Segun, 2021), socio-emotional capacities: observing etiquettes of communication to avoid misinterpretation and misunderstanding (Ng, 2012), collaboration with AI: working with AI as a co-participant where AI and users are learning from and about each other (Ng et al., 2024), and critical evaluation of AI technologies: discern inaccuracies and biases in AI tools and outputs (Ng et al., 2021).

### GenAI literacy in EFL writing

GenAI literacy is similar to AI literacy since most definitions of AI literacy are based on the use of GenAI tools such as ChatGPT. Nevertheless, because GenAI relies on the interplay of big data, algorithms and large language models (LLMs), GenAI literacy attaches more importance to high-order competences like criticality in evaluating how GenAI tools reflect ideologies in their interfaces and outputs, how GenAI-generated content reproduces misinformation, biases and stereotypes, and how the LLM-based algorithms privilege certain languages and types of knowledge over others (Darvin, 2025). Such critical thinking distinguishes GenAI literacy from a practical and use-centered orientation in most conceptualizations of AI literacy (Wang and Wang, 2025).

Accordingly, Warschauer et al. (2023) propose five concepts for (Gen)AI literacy in L2 writing: understand, access, prompt, corroborate, and incorporate. Firstly, students should understand the strengths, weaknesses, functions, and biases of AI tools for writing. In other words, students should know the answers to such questions as

“why AI is useful for writing, what it can do or cannot do and how to use it.”

Secondly, students should access AI functionalities for specific writing assignments. Students should consider the contextual factors of their writing, such as purposes, readers, and genres, and match their writing needs with AI functionalities including corpus search, text modification and paraphrasing and summary. Thirdly, students should be able to prompt AI to generate content or feedback on their writing. This ability requires L2 proficiency as students may need to construct and refine their prompts repeatedly until they get satisfactory AI-generated outputs. The process of seeking AI feedback is often iterative,

“moving from general to specific or from addressing one specific point to another.” (Wang and Wang, 2025, p. 6)

In addition, specific prompting strategies like few-shot, chain-of-thought, interactive, and dialogue-oriented prompting are also necessary skills for eliciting high-quality, sophisticated and reflective responses from AI (Park, 2025). Fourthly, students should corroborate the accuracy of AI-generated content for their writing against other sources of information. AI tools are sometimes known for their hallucinations due to probabilistic reasoning inherent in LLMs (Martin and Graulich, 2024). It means that they may produce seemingly logical and coherent texts containing false studies, inaccurate information and made-up references (Walter, 2024). Fifthly, students should incorporate AI-generated content in their writing ethically, acknowledging their use of AI openly and truthfully. Such an ethical practice is an essential part of applying AI tools (Chen et al., 2025), as AI writing tools have changed their roles from editors or evaluators to authors or co-authors (Su et al., 2023). This also requires students to know institutional policies on using AI for academic writing and to distinguish between plagiarism (e.g., copying and pasting without citations), patchwriting (e.g., replacing original words with synonyms), and intertextuality (e.g., referring to and quoting other texts openly).

By the same token, Wang and Wang (2025) propose four concepts for critical (Gen)AI literacy of EFL writers using ChatGPT: critical awareness of AI, critical positionality, critical strategies for interacting with AI and critical evaluation of AI affordances. Critical AI awareness means that EFL writers should be aware of coded biases and underrepresented languages resulting from GenAI's training data and employed algorithms. For example, ChatGPT's outputs may reproduce and thus reinforce the already dominant Western language ideology (Nee et al., 2022) and marginalize other rhetorics and languages. Such a consequence is called linguistic neo-imperialism (Lai, 2021) and language AI-nization (i.e., AI as a language colonization tool; Wang, 2025). Critical AI positionality means that EFL writers should maintain their originality and authorial voices while using GenAI for writing, because over-reliance on GenAI may diminish their autonomy and agency (Wang, 2025), and complicate authorship and their roles in knowledge building (Ko and Song, 2025). For instance, students in an advanced English writing course are found to only use AI for language aspects (grammar and spelling) rather than cognitive skills (content and organization; Hwang et al., 2025).

“Grammar and vocabulary were two common aspects for which the students sought ChatGPT's help.” (Wang and Wang, 2025, p. 6)

Their critical positionality also reflects their awareness that GenAI may not be

“accepted as credible sources of truth or epistemic authority” (Tang and Cooper, 2025, p. 732)

and that GenAI may deprive them of learning opportunities by providing shortcuts to knowledge (Barrot, 2023). Critical strategies for interacting with AI means that EFL writers should continuously adapt to GenAI's responses and adjust their prompting strategies to obtain accurate and targeted responses (Cain, 2024). Such a process is referred to as prompt engineering, which

“involves an iterative process of crafting instructions to guide GenAI in creating content and refining these instructions to produce more specific and relevant output.” (Park, 2025, p. 8)

Through this back-and-forth critical process of resisting outputs and refining prompts, users and AI are learning from and about each other, thus co-establishing the epistemic authority and sharing agency in creating knowledge. Critical evaluation of AI affordances means that EFL writers should evaluate GenAI's outputs in terms of information accuracy, linguistic and rhetorical conventions and contextual relevance. For instance, student writers may find ChatGPT good at replicating sophisticated vocabulary and generic structures but poor at conveying human emotions and personal experiences. Consequently,

“many responses from ChatGPT were homogeneous.” (Huang and Wang, 2025, p. 13)

Such a formal and rigid “AI-nized” style with a weak human touch is largely due to that fact that

“unlike humans, GenAI models cannot establish a connection to the extensional world as they lack access to the range of sensory inputs and embodied experiences that humans possess.” (Tang and Cooper, 2025, p. 738)

Accordingly, student writers are reported to select relevant ideas from AI outputs, rephrase them before incorporating them into a structured essay, which

“demonstrates higher-order thinking skills such as analyzing, evaluating, and synthesizing.” (Huang and Wang, 2025, p. 8)

Although these concepts describe various elements of (Gen)AI literacy in EFL/L2 writing, they remain fragmentary, mainly focusing on cognitive and behavioral perspectives. Therefore, it is necessary to have holistic frameworks of GenAI literacy in EFL writing, which not only encompasses students' mental and physical competencies, but also their emotional states and social responsibilities.

## Theoretical foundations

### Theories of literacy studies

There are two types of views on literacy: cognitive perspectives and sociocultural perspectives. Cognitive perspectives view literacy as a set of cognitive and linguistic processes and skills in reading and writing (Allington, 2002), autonomous of the social context in which they occur and the use to which they are put. From cognitive perspectives, AI literacy would only focus on users' internal and mental processes without considering the social contexts of AI use. Specifically, AI literate users understand the functions, interfaces and outputs of AI tools, and compose prompts for AI to generate content. However, they don't have to articulate motivations to use AI, identify biases and inaccuracies in AI, or consider ethics and risks in using AI, all of which depend on users' ideological, cultural and social contexts. This cognition-centered model is problematic because human mind is not mainly developed by abstract concepts but by actual experiences (Kolodner, 2006) in the material and social world. These experiences are not in the form of abstract concepts but linked with behaviors, perceptions, motivations, and feelings (Gee, 1992). Thus, there is a shift in literacy studies from cognitive perspectives to considering literacies as social practices (Mills, 2016).

In contrast, sociocultural perspectives view literacy not as abstract and cognitive processes in the private minds of individuals but as concrete social practices of reading and writing in the world of experience. Literacy is socially constructed through linguistic interactions among people, highlighting contextualized use of language within social events or practices (Street, 1984). Thus, literacy practices must be interpreted in relation to social and cultural factors such as material objects, political and economic conditions, social structures, power relations and cultural ideologies (Street, 1999).

From sociocultural perspectives, AI literacy would also be socially constructed, extending beyond technical knowledge and language skills of using AI as a mere technological instrument for checking grammar and spelling or generating content (Li, 2025). It would also involve competence to use knowledge about social contexts (e.g., task requirements, cultural norms, facilitating conditions, and academic conventions) to effectively, critically and responsibly engage in interaction with AI as a co-participant. Specifically, AI literate users can draw on their knowledge of task requirements and AI affordances to form perceptions of AI performances or usefulness (i.e., to what extent AI can accomplish certain tasks). They can also reflect on their own ability and facilitating conditions (e.g., access, cost, and assistance) to assess the efforts taken to use AI (i.e., how easy it is for them to use AI tools). They can fact-check AI content against other sources of information. They can utilize their prior knowledge to critically evaluate AI facilities and outputs (e.g., unequal access, social stratification, biased designs, and hallucinations) and understand algorithmic causes and social consequences of AI misrepresentations. They can strategically use AI-generated content while maintaining their own authorial voices according to social and ethical conventions.

In summary, cognitive perspectives view literacy as a set of cognitive and linguistic skills for reading and writing, which exists in an individual's mind without social contexts. However, sociocultural perspectives define literacy as a set of communicative competences, which is developed and utilized collectively through social interactions. As Robinson (1987) says,

“reading and writing are not unitary skills nor are they reducible to sets of component skills falling neatly under discrete categories (linguistic, cognitive); rather, they are complex human activities taking place in complex human relationships.” (p. 329).

Therefore, literacy studies have broadened the focus from cognitive and linguistic skills in an individual's mind to encompassing communicative competences in social and cultural interactions.

A similar shift of focus also happens in AI literacy studies. AI literacy is no longer just knowledge of AI technologies and language skills in using AI tools. Instead, it involves various social and cultural factors necessary for meaningful human-AI interactions, such as perception, attitude, usage, reflexivity, criticality, adaptability, and responsibility. Accordingly, it is necessary to adopt a holistic approach to AI literacy studies from affective, cognitive, behavioral and ethical perspectives, which

“provide a comprehensive framework for examining students' wider skill set of AI literacy.” (Ng et al., 2024, p. 1085).

### Theories of AI literacy in general

Most conceptualizations of AI literacy are based on Bloom's taxonomy of educational objectives as a theoretical foundation (Carlous et al., 2023), because it is

“a classic pedagogical theory that establishes the core foundation of AI taught to young learners.” (Ng et al., 2021, p. 4).

Specifically, Bloom's taxonomy consists of six components in a simple-to-complex hierarchical structure: knowledge, comprehension, application, analysis, synthesis and evaluation (Bloom, 1956). Bloom's taxonomy focuses on cognitive competences and skills, but ignores cultural and social differences. Since literacy is constructed within social and cultural contexts, it is necessary to involve a socio-cultural perspective in the theoretical frameworks of AI literacy studies.

Accordingly, Ng et al. (2021) propose a theoretical framework for AI literacy, with four dimensions: know and understand, use and apply, evaluate and create, and ethics, which are

“inspired from the cognitive domains in Bloom's taxonomy.” (p. 2).

Specifically, know and understand AI means knowing the basic functions of AI and how to use AI applications. Use and apply AI means applying AI knowledge, concepts, and applications in different scenarios. Evaluate and create AI refers to high-order thinking skills (e.g., evaluate, appraise, predict, and design) with AI applications. AI ethics refers to human-centered considerations (e.g., fairness, accountability, transparency, ethics, and safety). There is clear correspondence between the first three aspects of this AI literacy framework and the cognitive domains of Bloom's taxonomy. Namely, know and understand AI matches knowledge and comprehension. Use and apply AI is mapped onto application. Evaluate and create AI corresponds to analysis, synthesis and evaluation. As for AI ethics, AI literate students should understand social and cultural contexts and become responsible and ethical users in minimizing potential harm of misusing AI systems and content and promoting equitable access to AI applications in society (Usher and Barak, 2024).

Three years later, Ng et al. (2024) expand their framework which not only has cognitive and ethical dimensions as explained before, but also affective and behavioral dimensions. The rationale for this expansion is

“to further develop a deeper understanding of students' attitudes toward AI and their socio-emotional competencies, and to encourage collaboration, engagement and problem-solving.” (p. 1085).

Indeed, a holistic approach to AI literacy should include not only technical aspects but also socio-emotional capacities and high-order thinking skills (Biagini, 2024). Specifically, affective dimension refers to intrinsic motivation, self-efficacy, career interest and confidence in learning AI, the four of which determine the attitude toward AI. Behavioral dimension refers to behavioral intention, engagement and collaboration to facilitate group work and communication in AI environments.

However, the above theoretical frameworks are designed for AI literacy in general without considering disciplinary features. But (Gen)AI literacy is a context-bound notion that is shaped by the affordances and limitations of AI tools, and users' knowledge, skills, and perceptions of AI algorithms and outputs in specific domains (Liu et al., 2025). Therefore, disciplinary specificities should be considered (Ng et al., 2021) in studying domain-specific AI literacy (Bozkurt, 2024). Considering the increasing use of (Gen)AI in the domain of EFL writing, researchers have recently developed theoretical frameworks reflecting AI literacy of EFL student writers (Huang et al., 2025; Xu et al., 2025).

### Theories of (Gen)AI literacy in EFL writing

Xu et al. (2025) propose a four-dimensional framework for assessing Chinese university students' AI literacy in L2 writing: understanding, use, evaluation and ethics. Understanding means students' knowledge of and attitudes to AI tools. Use means how students apply AI tools during L2 writing process. Evaluation refers to how students analyze, compare, select, and critically evaluate AI tools and their generated content. Ethics focuses on students' awareness of (un)ethical conducts and potential risks in using AI for L2 writing.

Similarly, Huang et al. (2025) also propose a framework for assessing AI literacy in L2 writing among Chinese university students. It also has four dimensions: understanding, application, ethics and risk awareness, and self-efficacy and attitudes. Specifically, understanding means that learners understand types and functions, (dis)advantages, and future applications of AI tools for L2 writing. Application means that learners use AI tools to enhance L2 writing skills (e.g., vocabulary diversity, grammatical accuracy, and paragraph structure). Ethics and risk awareness means that learners consider ethical implications and potential risks of using AI (e.g., plagiarism and privacy breach) and distinguish between AI generated content and human writings. Self-efficacy and attitudes means that learners are confident and capable of using AI in L2 writing, trust and accept AI feedback, and believe AI can reduce stress and anxiety, and make the writing process interesting and enjoyable.

It can be seen that the first three dimensions (i.e., understanding, application, ethics, and risk awareness) are classic facets of previous AI literacy frameworks (Ng et al., 2021; Xu et al., 2025). But the other dimension (i.e., self-efficacy and attitudes) is unique to this framework. According to Huang et al. (2025), the reason for including self-efficacy and attitudes as a separate dimension is that it enables

“a more systematic account of learners' motivational beliefs, affective dispositions, and sustained-use intentions in AI-assisted writing.” (p. 19).

Therefore, the key concept here is intention for

“successful long-term and sustainable use of AI.” (Carlous et al., 2023, p. 4).

Indeed, self-efficacy (i.e., people's belief in their ability) directly shapes motivation and behavioral choice (Bandura, 1997), and is shown to influence EFL learners' behavioral intention, confidence, frequency and persistence of using AI tools in writing tasks (Selim, 2024). Attitude involves such affective factors as curiosity, trust, anxiety, or resistance (Carolus et al., 2023) and influences EFL learners' intention to adopt technologies (Biagini, 2024). EFL learners with positive attitudes to AI and high self-efficacy/confidence in using AI tend to have a strong intention/motivation to use AI tools to improve L2 writing (Teng, 2024; Wu et al., 2024). Simply put, self-efficacy and attitudes in the framework by Huang et al. (2025) both point to EFL learners' intention to use AI in writing.

A comparison of the two theoretical frameworks of AI literacy for L2 writing shows that both of them cover cognitive, behavioral and ethical perspectives with similar dimensions: understanding, use/application, and ethics/ethics and risk awareness. However, Xu et al. (2025)'s framework (i.e., understanding, use, evaluation, and ethics) does not include an affective perspective (e.g., intention, attitude, emotion, perception, and self-efficacy) as a distinct dimension though

“Xu et al. integrate attitudinal orientation into the ‘understanding' dimension” (Huang et al., 2025, p. 19).

learner perception of AI's contribution to improving L2 writing. On the other hand, Huang et al. (2025)'s framework (understanding, application, ethics and risk awareness, and self-efficacy, and attitudes) lacks an explicit high-order cognitive dimension (e.g., critical evaluation) though its “risk awareness” indicates elements of criticality: risks of privacy breach, data misuse and overreliance on AI. But their framework includes an affective perspective (i.e., self-efficacy and attitudes). As explained earlier, this affective perspective, though encompassing many related factors (e.g., anxiety, curiosity, satisfaction, self-efficacy, confidence, trust, interest, and attitude), can be summarized as contributing factors to learner intention of sustainable use of AI in L2 writing. Similar views are expressed by Al-Sharafi et al. (2023), who find students' satisfaction with AI chatbots result in their intention to continue using chatbots in learning activities. Zou and Huang (2023) also find that Chinese doctoral students' positive evaluation and easy handling of AI technology enhance their affective reaction (e.g., excitement, interest, curiosity, trust, and satisfaction) and self-efficacy, resulting in their favorable attitude and increasing behavioral intention to use AI tools in English academic writing. Between attitude and intention, the former influences the latter. People with a favorable attitude toward technology are likely to have a strong intention to use it (Estriegana et al., 2019). Attitudes as part of dispositions influence motivation and intention toward behavior in AI literacy (Tagare et al., 2025). Moreover, according to a systematic literature review of GenAI literacy in schools by Park (2025), 82.3% of studies

“examined students' attitudes toward GenAI, focusing on curiosity, interest, and self-efficacy.” (p. 10).

Obviously, Park considers curiosity, interest and self-efficacy as different aspects of attitudes. Park also believes that

“attitudes toward GenAI are a critical component of GenAI literacy, significantly influencing students' engagement, motivation, and perceived competency in effectively employing these GenAI tools.” (p. 10).

Since Park treats engagement, motivation and competence in using GenAI tools as three equal dimensions influenced by attitudes, these three dimensions should be deemed as equal components of GenAI literacy, which correspond to application, intention and evaluation in a framework of GenAI literacy. In contrast, attitude as well as other affective factors (e.g., interest, curiosity, and self-efficacy) should be considered as antecedent determinants of intention in GenAI literacy.

Accordingly, synthesizing the above two theoretical frameworks will result in a new (Gen)AI literacy framework in EFL writing with five dimensions: understanding, intention, application, evaluation, and ethics. Understanding means students understand the underlying mechanisms, functions and limitations of GenAI tools for EFL writing (Park, 2025). Intention means students intend to learn about and adopt GenAI tools as a long-term assistant in EFL writing (Carlous et al., 2023). Application means students use GenAI tools to enhance EFL writing quality and skills (Hwang et al., 2025). Evaluation means students critically evaluate GenAI technologies and generated content during EFL writing (Yim, 2024). Ethics means students comply with ethical principles and realize potential risks of using GenAI tools for EFL writing (Farrokhnia et al., 2024).

Although the five dimensions of (Gen)AI literacy framework in EFL writing have been defined and explained in a linear fashion, this linear approach to literacy conceptualization may be

“failing to capture the interconnectedness of its different components.” (Wang and Wang, 2025, p. 14)

In fact, the five dimensions should form an iterative cycle with overlapping among them. For example, understanding and intention, though considered as the starting points of (Gen)AI literacy, are often strengthened by student writers' application of GenAI tools, which is also involved in an iterative cycle with evaluation, reflection and ethics (Ngo and Hastie, 2025).

### Pedagogical implications

With increasing presence of GenAI in university, “it would be almost impossible to prohibit GenAI usage among university students.” (Wang et al., 2024, p. 8).

University educators thus need to adjust their curriculums to integrate GenAI into different disciplines and teaching contexts. In terms of EFL writing, EFL teachers can therefore draw on the five dimensions of GenAI literacy framework, and redesign their writing pedagogies to support student writers in using GenAI tools effectively and ethically.

The first dimension is understanding of GenAI tools, including their underlying mechanisms, functions and limitations for EFL writing. EFL teachers, therefore, can introduce basic operational principles for LLMs of GenAI:

“embedding-based contextual representation of words, next-word prediction, and error correction-based learning.” (Goldstein et al., 2025, p. 2).

They can then explain how LLMs learn various patterns and relations among words, phrases and sentences from a huge database of language. The models then calculate probabilities for each possible word following an input sequence before predicting the most likely word.

“This process is then repeated, with each new prediction informing the subsequent predictions until the desired length of the generated text is achieved.” (Tang and Cooper, 2025, p. 738).

After familiarizing students with these underlying mechanisms, teachers can demonstrate various functions of GenAI for EFL writing. For example, GenAI tools like ChatGPT can interact with EFL student writers in real time, correct linguistic errors, generate original texts, and provide genre-specific patterns (Shen et al., 2025). ChatGPT's most notable affordance is providing instant, interactive, diagnostic, detailed and individual feedback on EFL students' writing (He and Wang, 2025). It saves them time waiting for teacher feedback and facilitates their autonomous and self-regulated learning (Crompton and Burke, 2023), as EFL student writers can exercise

“clear control over the chatbot by issuing commands, making revisions and managing writing process independently” (Huang and Wang, 2025, p. 13).

Meanwhile, EFL teachers should also alert students to the flip side of GenAI in writing. The most notable limitation is the biased or inaccurate content generated by GenAI. The reason is that LLMs rely on

“probabilistic patterns derived from pre-trained large datasets” (Park, 2025, p. 3).

which fundamentally are human data with social values, thus being subject to manipulation and bias.

The second dimension is intention to learn about and adopt GenAI tools as long-term assistants in EFL writing. This dimension is shaped by various affective factors such as perception, attitude, and self-efficacy. Accordingly, when EFL teachers demonstrate how GenAI provides personalized feedback on lexical, structural, and stylistic aspects of student essays (Barrot, 2023), students may develop stronger perceptions of its usefulness. Such perceptions can foster positive attitudes to GenAI, which in turn enhance their intention to adopt tools such as ChatGPT (Zou and Huang, 2023). In addition, teachers should design activities that train students to strategically use multiple affordances of GenAI across different phases of the writing process, including task interpretation, topic selection, information searching, structural planning, and essay revising. This scaffolded support that gradually increases in complexity can strengthen students' self-efficacy/confidence in L2 writing over time (Hyland, 2007). Higher self-efficacy has been shown to predict greater behavioral intention to use AI tools, thereby promoting sustained and confident engagement (Selim, 2024). Indeed,

“when individuals perceive they can successfully use AI tools, they tend to continue using them over time.” (Huang et al., 2025, p. 5).

The third dimension is application of GenAI tools to enhance EFL writing quality and skills. It covers using GenAI tools for pre-writing planning, improving linguistic accuracy and overall structure, and revising writings (Xu et al., 2025). In terms of writing quality, EFL teachers can demonstrate certain prompting strategies for eliciting detailed corrective feedback from GenAI to polish language and enhance global structure (Mizumoto et al., 2024), and draw comparison between multiple GenAI-revised versions. They can also highlight the importance of pre-writing planning, which contributes to fluent and efficient writing (Ellis, 2021). For example, writing assignments without a straightforward outline would compel students to use GenAI for brainstorming and organizing ideas prior to the writing process. Apart from improving writing quality, EFL teachers can also focus on how to use GenAI for skill-learning in L2 writing. For instance, teachers can prompt GenAI to provide a list of specific writing skills accompanied by model texts to enhance

“English writing skills relating to organizational structures, language and content.” (Liu et al., 2024, p. 18)

Another important skill in GenAI-assisted writing is synthesizing AI content with authorial voices to maintain originality and autonomy. Teachers can accordingly demonstrate several options: asking students to formulate their own ideas before using AI, prompting AI to generate an outline based on their freewriting, or selecting AI-generated ideas to get inspired before expanding them to represent their own voices (Wang and Wang, 2025). In this way, EFL students' application of GenAI for writing would shift from complete rejection or full acceptance to selective adoption (Hakim, 2025).

The fourth dimension is critical evaluation of GenAI tools and their generated content. It involves assessing the potentials and pitfalls of using AI in EFL writing, choosing suitable AI tools and functions for different writing tasks, verifying the authenticity and reliability of AI outputs against other sources, and comparing, analyzing and selecting AI generated content (Xu et al., 2025). All these critical competences depend on students' understanding of writing contexts, such as

“their self-assessment of language proficiency, their perception of what constitutes good writing, and their consideration of the intended audience.” (Wang and Wang, 2025, p. 11).

Therefore, EFL teachers need to emphasize the contextual factors of each writing task, such as writing purposes, communication media, target readers, generic, and rhetoric conventions (Warschauer et al., 2023). They can then demonstrate how these factors combined with knowledge of AI technologies and students' own attributes will determine the way EFL students evaluate and select AI tools and affordances, adapt and integrate AI outputs into their writing. Teachers' efforts in this dimension aim to promote students' consciousness and competence to review, revise and reflect in AI-aided writing (Escalante et al., 2023) and thus address a key pedagogical concern: students' use of AI tools and outputs without critical evaluation can hinder their language acquisition and learning autonomy (Barrot, 2023).

The fifth dimension is ethics of using GenAI tools for EFL writing. It involves student writers' ethical conducts for academic integrity and their awareness of social risks such as breaching privacy, using unauthorized materials and leaking sensitive information (Xu et al., 2025). EFL teachers can explain ethical principles and regulations on academic writing in general and EFL writing in particular. They can then

“analyze real-world cases of academic misconduct resulting from the misuse of AI tools” (Xu, 2025, p. 11).

enabling student writers to distinguish between plagiarism, patchwriting, and intertextuality. Afterwards, students are expected to work in groups, developing policies of ethics for their own use of GenAI in writing. In addition to academic integrity, teachers also need to remind students of their social responsibilities of using GenAI in writing, such as citing reliable sources, protecting intellectual property, promoting harmonious narratives and conveying factual information, in order to

“collectively build a safe, reliable and accountable community with responsible AI literate citizens.” (Xu et al., 2025, p. 13).

All these teaching efforts align with the pedagogical tradition of emphasizing the social and ethical dimensions of students' literacy practices (Giroux, 1997).

## Conclusion and future directions

This conceptual analysis investigates relevant literature on (Gen)AI literacy in EFL/L2 writing, clarifying conceptualizations of AI literacy, especially criticality of GenAI literacy. It concludes that GenAI literacy in EFL writing is not only basic understanding and technical skills of using GenAI, but also critical evaluation and ethical consideration about GenAI tools and outputs. It is argued that criticality, as the core of GenAI literacy, facilitates EFL writers' autonomy and agency amidst the increasing prevalence of GenAI in education. The analysis also traces the development of literacy theories from cognitive perspectives to sociocultural perspectives. Accordingly, theories of AI literacy encompass affective, cognitive, behavioral and ethical perspectives, reflecting the nature of AI literacy as socially-constructed competences. Specifically, the analysis synthesizes theoretical frameworks of (Gen)AI literacy in EFL/L2 writing, illustrating five key dimensions: understanding, intention, application, evaluation and ethics. For practical significance, the analysis also explores pedagogical implications of GenAI literacy for EFL writing.

Future studies can use multiple data resources (e.g., questionnaires, interviews, and observations) and mixed methods (i.e., quantitative and qualitative approaches) to validate the dimensions of theoretical frameworks of GenAI literacy in EFL writing. Moreover, researchers can investigate how EFL students' demographics influence their GenAI literacy in writing, such as gender, age, educational level, English proficiency and cultural background. Finally, since writing quality is the ultimate result of EFL writing education, future research can examine how specific dimensions GenAI literacy predict various aspects of EFL writing quality such as lexical diversity, syntactic complexity and logical organization. Longitudinal studies can capture how GenAI literacy development correlates with improvement in EFL writing capacity.
